# Non-natural amino acids into LfcinB-derived peptides: effect in their (i) proteolytic degradation and (ii) cytotoxic activity against cancer cells

**DOI:** 10.1098/rsos.221493

**Published:** 2023-06-14

**Authors:** Diego Sebastián Insuasty-Cepeda, Andrea Carolina Barragán-Cárdenas, Natalia Ardila-Chantre, Karen Johanna Cárdenas-Martínez, Isabella Rincón-Quiñones, Yerly Vargas-Casanova, Alejandra Ochoa-Zarzosa, Joel Edmundo Lopez-Meza, Claudia Marcela Parra-Giraldo, Luis Fernando Ospina-Giraldo, Ricardo Fierro-Medina, Javier Eduardo García-Castañeda, Zuly Jenny Rivera-Monroy

**Affiliations:** ^1^ Chemistry Department, Universidad Nacional de Colombia, Bogotá, Carrera 45 No 26-85, Building 451, office 409, Bogotá 11321, Colombia; ^2^ Biotechnology Institute, Universidad Nacional de Colombia, Bogotá, Carrera 45 No 26-85, Bogotá 11321, Colombia; ^3^ Pharmacy Department, Universidad Nacional de Colombia, Bogotá, Carrera 45 No 26-85, Building 450, Bogotá 11321, Colombia; ^4^ Microbiology deparment, Pontificia Universidad Javeriana, Bogotá, Carrera 7 No 40-62, Building 450, Bogotá 11321, Colombia; ^5^ Multidisciplinary Centre for Studies in Biotechnology, Universidad Michoacana de San Nicolas de Hidalgo, Km 9.5, Morelia, Mexico

**Keywords:** non-natural amino acids, LfcinB-derived peptides, cytotoxic activity, MCF-7 cells, proteolytic degradation

## Abstract

The dimeric peptide ^26^[F]: (RRWQWR**F**KKLG)_2_-K-Ahx has exhibited a potent cytotoxic effect against breast cancer cell lines, with position 26 (F) being the most relevant for anti-cancer activity. In this investigation, six analogues of the ^26^[F] peptide were synthesized in which the 26th position was replaced by non-natural hydrophobic amino acids, finding that some modifications increased the resistance to proteolytic degradation exerted by trypsin or pepsin. Additionally, these modifications increased the cytotoxic effect against breast cancer cells and generated cell death mediated by apoptosis pathways, activating caspases 8 and 9, and did not compromise the integrity of the cytoplasmic membrane. Finally, it was found that the modified peptides have a broad spectrum of action, since they also have a cytotoxic effect against the HeLa human cervical cancer cell line. Peptide ^26^[F] was inoculated in mice by ip administration and the lethal dose 50 (LD_50_) was between 70 and 140 mg kg^−1^. While for the ^26^[1-Nal]: (RRWQWR-1-Nal-KKLG)_2_-K-Ahx peptide, a dose-response test was performed, and the survival rate was 100%. These results suggested that these peptides are safe in this animal model and could be considered as promissory to develop a treatment against breast cancer.

## 1. Introduction

Cancer remains a public health problem worldwide, being the second leading cause of death after cardiovascular disease [1]. Among all types of cancer, breast cancer represents a major health challenge in the world, since it is the most diagnosed today, with 2.26 million cases in 2020, and caused almost 685 000 deaths among women worldwide [2]. Currently, the main approaches to treating this disease are systematic therapies such as chemotherapy and endocrine therapy, as well as localized therapies such as radiation therapy and surgical resection. These treatments are of great importance, since they have an efficacy of between 25% and 40% and manage to reduce the risk of recurrence after 5 years to below 5% in the early stages. However, they are not targeted treatments and have low selectivity, causing side effects such as hot flashes, oedema, asthenia, myalgia, neutropenia, fractures, alopecia and new cancers, among many others, which significantly impair patients' self-esteem and quality of life [3]. Therefore, it is necessary to look for new treatments that could complement conventional ones, ones that would be more selective and therefore generate fewer side effects.

As new therapeutic agents, antimicrobial peptides (AMPs) have gained special attention in recent decades. AMPs are peptides that are less than 100 amino acids in length and are part of the innate immune system. Some AMPs are positively charged molecules with cationic and hydrophobic residues and amphipathic properties that can act on bacteria through membranolytic and non-membranolytic mechanisms [4]. AMPs have been found to have other biological functions, such as immunomodulatory, antifungal, antiviral and anti-cancer activity, among others [5–8]. Given the high number of AMPs that also exhibit anti-cancer activity, a new classification has emerged known as anti-cancer peptides (ACPs). ACPs are selective for malignant cells and act on plasma membrane receptors and/or intracellular targets, giving them a broad spectrum of action against different types of cancer [9]. Bovine lactoferricin (LfcinB) is a 25-amino acid ACP that is generated from the enzymatic digestion exerted by gastric pepsin of the bovine lactoferrin protein. LfcinB has been found to have immunomodulatory, antibacterial and anti-cancer activity in colon, leukemia, lung and breast cell lines, among others [10–12].

Solid-phase peptide synthesis has allowed chemical and structural modifications to be made to ACPs in order to enhance their activity and increase their resistance to proteolytic degradation [9]. Cyclic, dimeric and tetrameric peptides containing short sequences derived from LfcinB have been shown to exhibit 5- to 10-fold greater antibacterial or anti-cancer activity than LfcinB [13–16]. The dimeric peptide LfcinB (20–30)_2_: (^20^RRWQWRMKKLG^30^)_2_-K-Ahx has been shown to have a cytotoxic effect against MDA-MB-468 and MDA-MB-231 breast cancer cell lines, with IC_50_ values of 5 and 14 µM, respectively [15]. The 26th residue of this dimeric peptide (Met) plays a very important role in the anti-cancer activity: when the residue ^26^Met was changed to Lys or Asp, the anti-cancer activity was lost, while when it was replaced by hydrophobic amino acids such as Leu or Phe, the cytotoxic effect against MDA-MB-468 and MCF-7 breast cancer cells significantly increased [16]. In addition, the (RRWQWRFKKLG)_2_-K-Ahx peptide (^26^[F]) exhibited significant anti-cancer activity in colon cancer cell lines CaCo-2 and HTC-116 [17]. These results suggest that the higher the hydrophobicity of the 26-position amino acid, the greater the cytotoxic effect it has on cancer cell lines. Taking this into account, in the present investigation we wanted to evaluate whether increasing the hydrophobicity of this position could increase the cytotoxic effect in cancer lines. Dimeric peptides derived from (^20^RRWQWRFKKLG^30^)_2_-K-Ahx, containing at 26th position hydrophobic nonnatural amino acids such as diphenylalanine (Dip), 1-naphthyl-phenylalanine (1-Nal), 4-benzoyl-phenylalanine (Bpa), among others, were synthesized, and their cytotoxic effect against cancer cells as well their resistance to proteolytic degradation were evaluated.

## 2. Material and methods

### 2.1. Reagent and materials

Rink amide resin, Fmoc-Arg(Pbf)-OH, Fmoc-Trp(Boc)-OH, Fmoc-Gln(Trt)-OH, Fmoc-Phe-OH, Fmoc-Lys(Boc)-OH, Fmoc-Leu-OH, Fmoc-Gly-OH, Fmoc-6-Ahx-OH, Fmoc-Lys(Fmoc)-OH, Triton-X, piperidine, N,N-dimethylformamide (DMF), dichloromethane (DCM), dicyclohexylcarbodiimide (DCC), 1-hydroxy-6-chloro-benzotriazole (6-Cl-HOBt), ninhydrin, potassium cyanide (KCN), ethanol, pyridine, phenol, trifluoroacetic acid (TFA), ethyl ether, triisopropylsilane (TIS), ethanedithiol (EDT), acetonitrile (ACN), methanol (MeOH) and solid phase extraction columns Supelco, Trypsin-EDTA solution and pepsin 2500 units mg^−1^ were purchased from Sigma-Aldrich (St Louis, MO, USA). The non-natural amino acids diphenylalanine, 1-naphthyl-phenylalanine, 4-benzoyl-phenylalanine, homophenylalanine (hF), 4-(Fmoc-amino) benzoic acid (4-Abz) and 2-(Fmoc-amino) benzoic acid (2-Abz) were obtained from AAPPTec (Louisville, KY, USA). The cell lines MCF-7, HeLa, HEK-293 were purchased from ATCC (Manassas, VA). The primary cell culture of fibroblasts was obtained from the foreskin by the Laboratory of Cellular Physiology of the National University of Colombia, and the fibroblasts were cultured and frozen. Some vials were thawed and cultured for up to seven passes, since no transformation/immortalization was done. Annexin V and Alexa Fluor 488 conjugate was purchased from Invitrogen (Eugene, Oregon). The DMEM culture medium and trypsin were obtained from Sigma-Aldrich (St Louis, MO, USA). Bovine fetal serum was purchased from Gibco (Waltham, MA, USA). Calcium assay kit and Fluorescein CaspGLOW Active Caspase-8 or Caspase-9 Staining Kit were obtained for BD Biosciences (San Diego, CA, USA) and Invitrogen Thermo Fisher Scientific (Waltham, MA, USA), respectively.

### 2.2. Peptide synthesis (SPPS-Fmoc/tBu)

The peptides were obtained by manual solid phase synthesis, using the Fmoc-tBu strategy (SPPS-Fmoc/tBu) in accordance with Vergel *et al.* [18]. Rink amide resin (0.1 g) was swollen with DMF for 1 h at room temperature (RT). The Fmoc group removal was carried out by treating resin or resin-peptide with 5% piperidine, 0.1% triton X-100 in DMF, and under constant stirring for 15 min at RT (three times). The Fmoc-amino acid was activated by dissolving Fmoc-AA/DCC/6-Cl-HOBt (1 : 1 : 1 equivalent and five excess with respect to the substitution resin) in DMF, and the reaction mixture was gently stirred for 15 min at RT. After the reaction mixture was mixed with the resin or resin-peptide and gently stirred for 12 h at RT, the solution was eliminated by filtration and the resin or resin-peptide was washed with DMF (twice), isopropyl alcohol (twice) and DCM (twice). The protecting groups on the side chains were removed, and the peptide was detached from the solid support, treating the resin-peptide with cleavage cocktail TFA/EDT/TIS/H_2_O (92.5/2.5/2.5/2.5% w/w), at a ratio of 1 : 10 w/v. The reaction mixture was stirred for 8 h, and subsequently it was filtered, the solution was treated with ethyl ether, and the solid was washed with ethyl ether five times.

### 2.3. RP-SPE purification

The peptides were purified by means of solid phase extraction in reverse phase mode (RP-SPE) [19]. The crude peptide was dissolved in water (1 mg ml^−1^) and analysed via RP-HPLC. Based on the chromatographic profile, an elution programme was designed for the purification of up to 100 mg of peptide by RP-SPE. The percentage of organic modifier (ACN) was progressively increased, and the fractions (15 ml) containing the pure peptide were collected, lyophilized and stored at −20°C.

### 2.4. RP-HPLC analysis

The peptides were analysed using a Hitachi Primaide-DAD 1110 chromatograph and a Chromolith RP-18e High Resolution column (100 × 4.1 i.d. mm) (Merck, Darmstadt, Germany). Water containing 0.05% TFA was used as solvent A, and ACN containing 0.05% TFA was used as solvent B. The elution programme used was 5/5/50/100/100/5/5% B at 0/1/9/13/13.1/15 min at a flow rate of 2 ml min^−1^ and a wavelength of 210 nm. Ten microlitres of each peptide solution was injected at an approximate concentration of 1 mg l^−1^.

### 2.5. High-resolution ESI-Q/TOF MS characterization

Two microlitres of each pure peptide solution was analysed in a Bruker Impact II LC Q-TOF MS equipped with electrospray ionization (ESI) in positive mode. The chromatographic conditions were: intensity Solo C18 column (2.1 × 100 mm, 1.8 µm) (Bruker Daltonik), at a temperature of 40°C and a flow rate of 0.250 ml min^−1^. Solvent A water and solvent B ACN, each containing 0.1% formic acid, were used as the mobile phase. The elution programme was 5/5/95/95/5/5% B at 0/1/11/13/13.1/15 min. ESI source conditions: end plate offset 500 V, capillary 4500 V, nebulizer 1.8 bar, dry gas nitrogen 8.0 L min^−1^, dry temp 220°C. Scan mode AutoMS/MS with spectral range 20–1000 *m/z*, spectra rate 2 Hz and collision energy of 5.0 eV.

### 2.6. Cell culture

For all cell lines, the medium used was Dulbecco's Modified Eagle's Medium (DMEM)/Nutrient Mixture F-12 Ham and supplemented with 10% fetal bovine serum (SFB), and 1.5 g l^−1^ NaHCO_3_ and NaOH were added up to pH 7.4, amphotericin (200 µg ml^−1^), and 1% penicillin and streptomycin. For the primary culture cells of fibroblasts, in addition to the above, hydrocortisone (250 µg ml^−1^) was added. All media were filtered through a 0.22 µm membrane.

### 2.7. MTT viability assays

**Figure 1.**
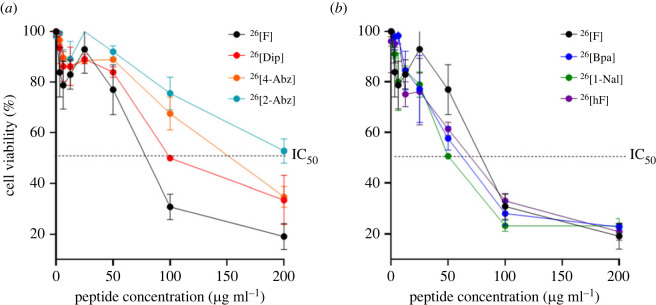
Cytotoxic effect of dimeric peptides against breast cancer cells. Left: cell viability plots of dimeric peptides with lesser cytotoxic effect than the original peptide ^26^[F] (black); ^26^[Dip] (red), ^26^[4-Abz] (orange), ^26^[2-Abz] (green). Right: cell viability plots of dimeric peptides with greater cytotoxic effect than the original peptide; ^26^[Bpa] (blue), ^26^[1-Nal] (green), ^26^[hF] (violet). The data represent the mean ± s.e. Three independent experiments with *n* = 4 each. (ANOVA, Sidak's multiple comparisons test was used, *p* ≤ 0.05).

The MTT assay was performed in accordance with previous reports [20]. Briefly, the cells were seeded with a complete medium in 96-well plates at a rate of 10 000 cells and 100 µl per well, and adhesion to the plates was allowed for 24 h. Later, the complete medium was removed, and an incomplete medium was added for synchronization for another 24 h. The cells were then incubated at 37°C for 2 h with 100 µl of peptide at the evaluated concentrations (200, 100, 50, 25, 12.5 and 6.25 µg ml^−1^). Next, the peptide was removed from the well and 100 µl of incomplete medium with 10% MTT was added and incubated for 4 h. The medium was replaced with 100 µl of isopropanol, and after 30 min of incubation at 37°C, the absorbance was measured at 575 nm. As a negative control, an incomplete culture medium with 10% MTT was used, and as a positive control, cells without MTT treatment were used. The IC_50_ value was determined from figure 1 (peptide concentration versus per cent cell viability) using a nonlinear regression model with variable slope and GraphPad Prism 9 statistical analysis software.

### 2.8. Trypsin degradation assays

Trypsin (20 μg) was dissolved in 450 µl of the buffer supplied by the manufacturer, and 50 µl was added to each peptide solution, maintaining a protease : peptide ratio of 1 : 100 (w/w). The samples were incubated at 37°C for 24 h. Aliquots were taken at 0, 5, 10, 15 and 20 min, the enzymatic reaction was stopped by adding 1 M HCl, and the mixture was analysed via RP-HPLC and LC-MS. Bovine serum albumin (BSA) and bovine lactoferrin (bLF) were used as the positive control, and the negative control was the peptide in buffer solution without trypsin.

### 2.9. Pepsin degradation assays

Pepsin was dissolved in 0.010 M HCl (pH 2.0). An aliquot of 50 µl was added to each peptide solution in a 1 : 100 (w/w) ratio. The peptides were dissolved in H_2_O-TFA 0.05% (v/v). This was incubated at 37°C for 3 h, aliquots were taken at 0 and 3 h, and the samples were heated for 5 min and then cooled at 4°C for the thermal inactivation of pepsin and characterized via HPLC and LC-MS. BSA and bLF were used as the positive control, and the negative control was peptide in buffer solution without trypsin.

### 2.10. Determination of the type of cell death (apoptosis/necrosis)

The cell death type assay was performed in accordance with previous reports [16]. The cells were seeded and synchronized in boxes of 24 wells at a concentration of 4 × 10^5^ cells per well in 400 µl per well, adhesion and synchronization were allowed, and the culture medium was replaced with medium containing the peptide to be evaluated and incubated for 2 or 24 h. Subsequently, the cells were harvested with trypsin, centrifuged at 2500 r.p.m. for 10 min, washed with PBS, and resuspended in 10 µl of staining buffer with the fluorochromes (10 mM Hepes pH 7.4; 10 mM NaCl and 2.5 mM CaCl_2_ containing 1 µl of PI fluorochrome and 1 µl of Annexin V). Cells with the fluorochromes were incubated at 37°C in the dark for 15 min and resuspended in 80 µl of staining buffer without fluorochromes for analysis via flow cytometry. The positive control for necrosis was cells treated with EDTA 15 mM for 60 min, and for apoptosis cells treated with actinomycin at 10 µM for 24 h. Negative control: cells without treatment.

### 2.11. K calcium efflux testing

The calcium release assay was performed in accordance with Jiménez-Alcántar *et al.* [21]. Cells (1 × 10^5^) were synchronized for 24 h in incomplete medium and then incubated with the dye indicator of the calcium assay kit (BD Biosciences) for 1 h. In a BD Accuri flow cytometer (BD Biosciences), a baseline reading was maintained for 1 min, and then the evaluated peptide solution was added at IC_50_ concentration and the reading was continued for an additional 4 min. 4-Alpha-Phorbol-12-myristate-13-acetate (3 mM, PMA, Sigma) was used as a positive control.

### 2.12. Caspase activity assay

The evaluation of caspases 8 and 9 was carried out in accordance with Jiménez-Alcántar *et al.* [21]. Cells (8 × 10^4^) were synchronized with incomplete medium for 24 h and then treated with the evaluated peptides at IC_50_ concentration. Subsequently, the cells were harvested and stained according to the manufacturer's instructions with the GaspGlow Fluorescein Active Caspase-8 or 9 staining Kit. Caspase activity was measured through the inhibitors LEHD-FMK (caspase 9 inhibitor) and IETDFMK (caspase 8 inhibitor) conjugated with fluorescein, which binds to the active enzyme. ActD (0.5 µM) was used as a death control.

### 2.13. Haemolytic assay

Haemolysis assays were performed in accordance with Cárdenas *et al.* [22]. The blood was donated by healthy volunteers with blood type O^+^. First, 5 ml of peripheral blood in EDTA was centrifuged at 500 r.p.m. for 15 min, and the erythrocytes were separated and washed three times with 0.9% saline solution. Then 100 µl of peptide solution (final concentrations of 200 at 6.2 µg ml^−1^) was mixed with 100 µl of erythrocytes (2% haematocrit) and incubated at 37°C for 2 h. It was then centrifuged at 2500 r.p.m. for 5 min and the absorbance of the supernatant was measured at 450 nm. Two controls were used; positive control: distilled water, and negative control: saline solution 0.9%.

### 2.14. Peptide toxicity *in vivo* model

*Experiment 1.* CD1 mice male (eight weeks old, weighing 33–35 g) were housed in groups of six animals per cage for each peptide ^26^[F] and ^26^[1-Nal]; three mice were inoculated by intraperitoneal via ip, and three mice were inoculated by subcutaneous via sc. Mice inoculated via ip or sc with saline solution were used as control. The peptide was dissolved in saline solution (7 mg ml^−1^) and the mice were inoculated only one time with 0.33–0.35 ml (dosage 70 mg kg^−1^) via ip or sc. *Experiment 2*. CD1 mice female (*n* = 4, eight weeks old, weighing 33–35 g) were inoculated only one time with peptide ^26^[F] (dosage 140 mg kg^−1^) or ^26^[1-Nal] (8.8 to 70 mg kg^−1^).

All the experiments were carried out in compliance with the guidelines and standards of Resolution 08430 of 1993 of the Colombian Ministry of Health and Law 84 of 27 December 1989 and were governed by the principles and standards for the care and use of animals, decreed by the institutional Ethics Committee from Science Faculty (Act 03-2018) of the National University of Colombia.

## 3. Results and discussion

### 3.1. Peptide obtention and their cytotoxic effect against MCF-7 cells

In previous studies, we have reported that divalent sequences derived from LfcinB exhibit a cytotoxic effect against cell lines derived from oral, breast and colon cancer [16,17,23]. The dimeric peptide (^20^RRWQWRFKKLG^30^)_2_-K-Ahx (coded here as ^26^[F]) was identified as promising, because it had a selective cytotoxic effect against breast cancer cell lines, and it was established that position 26 of the sequence is relevant for anti-cancer activity [16]. In addition, it was found that when position 26 of the sequence is occupied by hydrophobic amino acids, the anti-cancer activity is increased [16]. In order to enhance the cytotoxic effect of dimeric peptide ^26^[F], in this study, the phenylalanine residue was replaced by non-natural amino acids (table 1), which have greater hydrophobicity than Phe. Specifically, diphenylalanine (Dip), 1-naphtyl-phenylalanine (1-Nal), 4-benzoyl-phenylalanine (Bpa), 4-aminobenzoic acid (4-Abz), 2-aminobenzoic acid (2-Abz) and homophenylalanine (hF) were selected. In a manner similar to Phe, all selected non-natural amino acids have a benzene ring; amino acids Dip, Bpa and 1-Nal have an additional benzene ring, amino acid hF has an additional carbon, and finally, the incorporation of 2-Abz or 4-Abz in a peptide sequence allows having a benzene ring located within the peptide backbone. Our aims were to (i) determine if the increase in hydrophobicity at position 26 of the dimeric sequence causes an increase in anti-cancer activity against MCF-7 cells, and (ii) evaluate whether these substitutions generate resistance to proteolytic degradation of peptides caused by pepsin and trypsin. Gastric pepsin and trypsin are two of the main enzymes involved in metabolic barriers that make it difficult for drugs to reach their cellular target [24].

**Table 1.** Characterization of dimeric peptides.

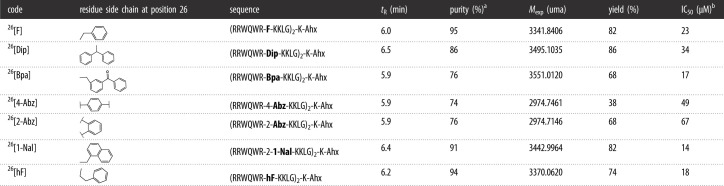

^a^Chromatographic purity at 210 nm.

^b^IC_50_ obtained in the MCF-7 breast cancer line with peptide treatment for 2 hours.

Six dimeric peptides, analogues of ^26^[F], were synthesized via SPPS-Fmoc/tBu, purified via RP-SPE, and characterized via RP-HPLC chromatography and ESI-QTOF mass spectrometry (table 1). For each peptide, the chromatographic profile showed a major specie, and the experimental mass determined by MS corresponded to the expected mass (see electronic supplementary material S1). As can be seen, peptides ^26^[1-Nal] and ^26^[hF] were obtained with higher chromatographic purity (above 90%) and yields (greater than 70%). The peptides ^26^[Dip], ^26^[Bpa], ^26^[2-Abz] and ^26^[4-Abz] were obtained with lower purity and yield, which may be attributed mainly to the difficulty of quantitatively completing the incorporation of the unnatural amino acid into the growing sequence during SPPS.

Regarding retention times (*t*_R_) in RP-HPLC analysis, peptides ^26^[1-Nal], ^26^[hF] and ^26^[Dip] exhibited the highest values, indicating that these peptides are more hydrophobic. Furthermore, peptides ^26^[2-Abz], ^26^[4-Abz] and ^26^[Bpa] showed *t*_R_ = 5.9 min, indicating that these peptides have a hydrophobicity similar to that of peptide ^26^[F]

The cytotoxic effect of the dimeric peptides against breast cancer cell line MCF-7 was evaluated (figure 1). As can be seen, the six peptides exerted a rapid and concentration-dependent cytotoxic effect against these breast cancer cells. Peptides ^26^[Dip], ^26^[4-Abz] and ^26^[2-Abz] exhibited a lower cytotoxic effect than the original peptide ^26^[F], with IC_50_ values of 34, 49 and 67 µM, respectively, while peptides ^26^[Bpa], ^26^[1-Nal] and ^26^[hF] exhibited a greater cytotoxic effect than the original peptide, with IC_50_ values of 17, 14 and 18 µM, respectively. It is important to note that peptides ^26^[1-Nal] and ^26^[hF] were of high purity (greater than 90%), close to the original peptide, and had IC_50_ values lower than the original peptide, suggesting that the hydrophobicity of position 26 of the sequence is relevant for anti-cancer activity against breast cancer lines (figure 1). The peptides obtained were soluble in aqueous medium (greater than 25 mg ml^−1^) which allowed toxicity studies to be performed in the murine model.

### 3.2. Pepsin and trypsin degradation studies

**Figure 2.**
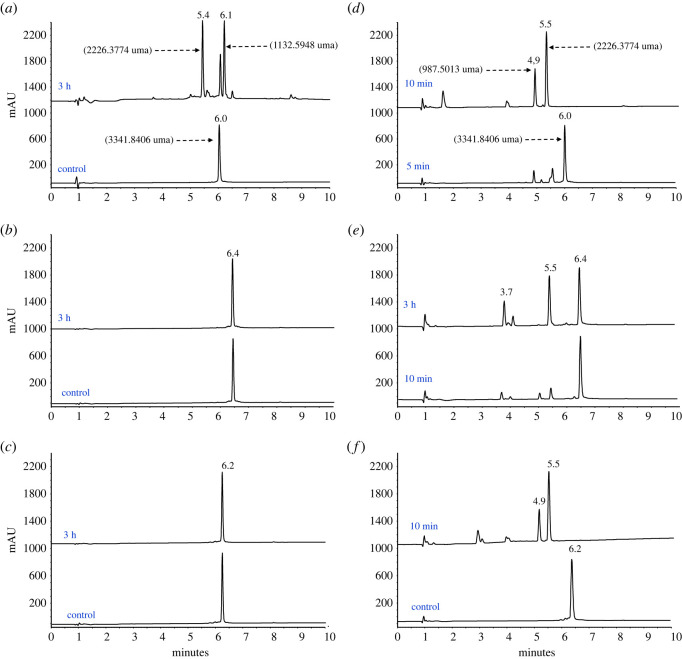
Pepsin and trypsin digestion of dimeric peptides. Pepsin digestion of: (*a*) ^26^[F], (*b*) ^26^[1-Nal] and (*c*) ^26^[hF]. Trypsin digestion of: (*d*) ^26^[F], (*e*) ^26^[1-Nal] and (*f*) ^26^[hF] The peptides were treated with pepsin for 3 h and trypsin for 5 min, 10 min and 3 h.

Dimeric peptides ^26^[F], ^26^[1-Nal] and ^26^[hF] were treated with pepsin or trypsin, in order to evaluate the proteolysis exerted by these enzymes (figure 2). The peptides were incubated with the enzyme and aliquots were taken at different times; then, the activity of the enzyme stopped, and the solutions were analysed via RP-HPLC and LC-MS. Untreated ^26^[F] peptide showed a chromatographic profile with a single species (*t*_R_: 6.0 min and 95% purity), and the ESI-MS spectrum showed signals at *m*/*z* relations corresponding to the [M + nH]^n+^ species, with n ranging from 4 to 9; the signals corresponded to the expected molecular weight 3341.8406 uma. When this peptide was treated with pepsin for 3 h, the chromatographic profile showed a peak of *t*_R_ = 6.0 min and two new species with *t*_R_ = 5.4 and 6.1 min (figure 2*a*). Using LC-MS, it was determined that the species with *t*_R_ = 5.4 min had a mass of 1132.5948 uma, which corresponded to the fragment RRWQWRF, while the other species with *t*_R_ = 6.1 min had a mass of 2264.3774 uma, corresponding to the other fragment, RRWQWRFKKLG-K(GLKK)-Ahx. In this case, the peptide was fragmented in two parts, and the cleavage only occurred in one branch at the N-terminal extreme of the ^26^Phe and C-terminal of ^25^Arg. These results are surprising, since multiple short fragments were expected to be generated, considering that pepsin cleaves sequences containing hydrophobic amino acids such as Trp, Phe and Tyr.

Similarly, when peptide ^26^[F] was treated with trypsin for 5 min, two fragments were also generated; the first corresponds to the sequence RRWQWR with a *t*_R_ = 4.9 min and a mass of 987.5013 uma, and the second was RRWQWRFKKKLG-K(FGLKK)-Ahx with a *t*_R_ = 5.5 min and a mass of 2226.3774 uma (figure 2*d*). These results suggest that trypsin hydrolyses the peptide bond between the carboxyl end of ^25^Arg and the N-terminus of ^24^Phe. In addition, similar to what was observed with pepsin, trypsin also produced only two fragments, and excision occurred on one branch of the dimer. Peptide ^26^[F] was more sensitive to trypsin than to pepsin, since the degradation products generated by trypsin were evident after 5 min of treatment, and when the peptide was treated with this enzyme for 10 min, the signal corresponding to the original peptide (*t*_R_: 6.0 min) disappeared completely (figure 2*d*).

The chromatographic profile of peptide ^26^[hF] treated for 3 h with pepsin exhibited a single peak, at *t*_R_ = 6.4 min. Similarly, the chromatographic profile of pepsin-treated peptide ^26^[1-Nal] also exhibited a single peptide, with *t*_R_ = 6.2 min. The chromatographic profiles of pepsin-treated and untreated peptides were similar, indicating that pepsin treatment for 3 h did not induce proteolytic degradation of either peptide (figure 2*b,c*). Treatment with pepsin showed that the ^26^[F] peptide was degraded into two fragments, while the ^26^[hF] and ^26^[1-Nal] peptides were unaffected, suggesting that the substitution of Phe by 1-Nal or hF, at 26th position of the dimers, induces resistance to proteolytic degradation caused by pepsin. These results support the notion that the pepsin recognition site is solely located at position ^26^F of the sequence. Substitution of this position with a non-natural amino acid disrupts the conformational interaction, thereby preventing the enzyme from acting at position 26 of the sequence. On the other hand, it is interesting to note that pepsin causes the degradation of the ^26^[F] peptide, because this sequence is part of LfcinB, which occurs in the digestion of bLF caused by gastric pepsin. The above suggests that the cyclic form of LfcnB may prevent the degradation of LfcinB caused by pepsin.

After treating peptide ^26^[hF] with trypsin for 10 min, similar to what was observed with peptide ^26^[F], two fragments were also generated: RRWQWR (*t*_R_ = 4.9 min) and RRWQWR-hF-KKKLG-K(FGLKK)-Ahx (*t*_R_ = 5.5 min) (figure 2*f*). These results suggest that trypsin cleaved the peptide bond at the C-terminal of ^25^Arg and the N-terminal of ^26^hF.

On the other hand, peptide ^26^[1-Nal] treated with trypsin for 10 min showed less degradation, with the majority being intact dimeric peptide (*t*_R_ = 6.4 min). When this peptide was treated with trypsin for 3 h, the chromatographic profile showed three main species, with *t*_R_ = 3.7, 5.4 min and unaffected peptide *t*_R_ = 6.4 min (figure 2*e*). The degradation pattern of peptide ^26^[1-Nal] (treated for 3 h) was similar to the degradation pattern of peptide ^26^[hF] when treated with the enzyme for 10 min. The findings suggest that resistance to proteolytic degradation is mainly due to steric impediment caused by the voluminous side chain of the amino acid 1-Nal, preventing the trypsin from cleaving the peptide chain between the C-terminal of ^25^Arg and the N-terminal of ^26^[1-Nal]; despite the fact that the latter is not directly related to the recognition site of trypsin.

Our results suggest that the dimeric structure confers resistance to proteolytic degradation, since it was observed that the enzymes only cut in a single branch of the dimer, and the two resulting fragments are preserved for up to 3 h. In addition, the inclusion of the non-natural hydrophobic amino acids 1-Nal or hF at position 26 prevents degradation caused by pepsin, while the inclusion of the amino acid 1-Nal slows down the enzymatic action of trypsin. The results suggest that dimerization of LfcinB-derived sequences is an efficient strategy, because (i) it potentiates the cytotoxic effect, (ii) it increases selectivity by non-tumorigenic cells, and (iii) it confers resistance to proteolytic degradation caused by digestive enzymes such as pepsin and trypsin, which tend to cause the most degradation to peptides and make oral administration route unfeasible, hence, most available oncologic peptides are administered subcutaneously or intravenously [25]. This is the first study of the resistance of dimeric peptides ^26^[F], ^26^[1-Na], ^26^[hF] against enzymatic degradation by trypsin or pepsin.

Our results are novel and interesting as they show that changing the amino acid at position 26, which is the cleavage site, changes the degradation pattern. The enzymes mainly act on a single arm of the dimeric structure and the fragments generated possess motifs with anti-cancer activity. Our results suggest that it is possible to design peptides that when degraded by enzymes generate active sequences, making this a powerful tool for the development of treatments.

### 3.3. Selectivity assays

**Figure 3.**
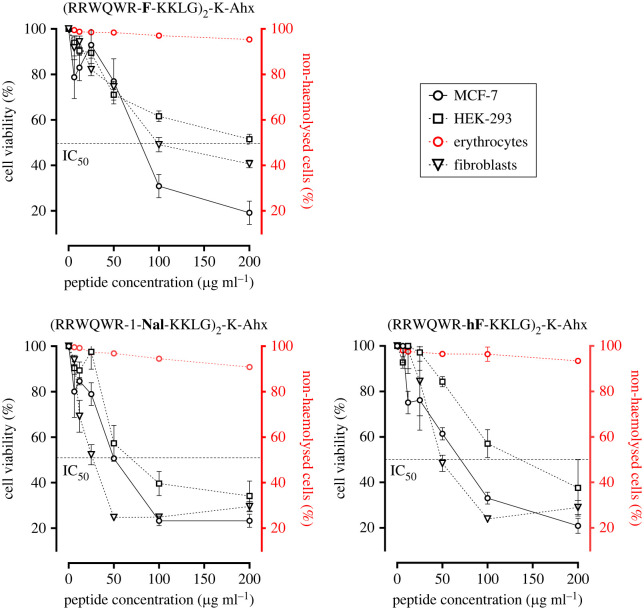
Cytotoxic effect of dimeric peptides against non-tumorigenic cells HEK-293 and erythrocytes. Breast cancer cells MCF-7 (black solid line ─○─); human kidney non-tumorigenic cell line HEK-293 (black dotted line ─□─); fibroblasts (black dotted line ─∇─) and erythrocytes (red solid line ─○─). The data represent the mean ± s.e. Three independent experiments with *n* = 4 each. (ANOVA, Sidak's multiple comparisons test was used, *p* ≤ 0.05).

To determine the *in vitro* selectivity, the cytotoxic effect of dimeric peptides ^26^[F]_,_^26^[Nal] and ^26^[hF] against breast cancer cells MCF-7, and three human non-cancer cell lines: (i) HEK-293 human kidney cell line, (ii) human primary fibroblasts, and (iii) human erythrocytes was compared (figure 3, table 2). The three peptides exerted a cytotoxic effect against fibroblasts, HEK-293 and MCF-7 cell lines in a concentration-dependent manner.

**Table 2.** Cytotoxic effect of the peptides over MCF-7, HeLa and HEK-293 cell lines, and human erythrocyte.

IC_50_ µg mL^−1^(µM)					
code	MCF-7	HeLa	fibroblasts	HEK-293	erythrocytes
^26^[F]	79(23)	24(7)	121(35)	203(59)	>200(>58)
^26^[1-Nal]	48(14)	55(16)	30(9)	86(25)	>200(>58)
^26^[hF]	60(18)	53(16)	63(19)	134(40)	>200(>59)

selectivity					
index IC_50_ no cancerous cells /IC_50_ MCF-7					
(index IC_50_ no cancerous cells/IC_50_ HeLa)					
code	HEK-293	fibroblasts	erythrocytes		
^26^[F]	2.6 (8.4)	1.5 (5.0)	>2.5 (>8.3)		
^26^[1-Nal]	1.8 (1.5)	0.6 (0.5)	>4.1 (>3.6)		
^26^[hF]	2.2 (2.5)	1.0 (1.2)	>3.3 (>3.7)		

The three peptides evaluated were highly selective with respect to erythrocytes, which are considered the gold standard for *in vitro* toxicity studies. As can be seen in table 2, peptide ^26^[F] showed the highest selectivity for breast cancer cells, since its IS was greater than 1.0 for the three cell lines. Followed by peptide ^26^[hF] which had an IS of 2.2 for HEK-293 but was not selective against fibroblasts with an IS close to 1.0. And finally, the peptide with the least selectivity was ^26^[1-Nal], finding the lowest IS values. It is noteworthy that peptides with unnatural modifications obtained IS values even lower than 1.0 for fibroblasts, indicating that these peptides affect this non-cancerous cell line to a greater extent than the cancer line. However, the results in erythrocytes mean that, despite the low selectivity in fibroblasts, the three evaluated peptides are promising.

To determine the toxicity of peptides ^26^[F] and ^26^[1-Nal] *in vivo* model, a preliminary study was carried out in CD1 male mice. The Irwin test was used to evaluate in qualitative manner the effect of peptide administration over motor functions, behaviour, physiology and pharmacological safety in rodents. This test is used as a first approach to study of the adverse effects generated by a pharmaceutical treatment in an *in vivo* model in preclinical test. Some authors suggest that initial dose could be between 100 and 300 times higher than the *in vitro* effective concentration [26], so for this study an initial dose equivalent to a 140 times higher concentration than IC_50_ value in MCF-7 cells was used.

Mice were inoculated using two administration routes: intraperitoneal (ip) and subcutaneous (sc) (table 3, experiment 1). With both peptides via sc, they showed similar behaviours; after 30 min, the mice showed decreased motor functions and manifestations of piloerection. After 24 h, all mice returned to their normal state and the survival rate was 100%. These results indicating that the peptide administration via sc is safe at the doses evaluated. In mice inoculated via ip (table 3, experiment 1) with peptides ^26^[F] or ^26^[1-Nal], motor function decreased 30 min after peptide administration and abdominal contortions were observed, possibly attributed to localized irritation. In addition, redness of the tail and ears was observed, which may be associated with peripheral vasodilation.

**Table 3.** Toxicity testing of peptides in mice.

peptide	survival (%)		mice number	dose (mg kg^−1^)
	experiment 1 (male mice)			
	ip	sc		
^26^[F]	100	100	3	70.0
control	100	100	3	0
^26^[1-Nal]	33	100	3	70,0
control	100	100	3	0
	experiment 2 (female mice)			
	ip			
^26^[F]	0		4	140.0
control	100		4	0
^26^[1-Nal]	100		3	70.0
	100		3	35.0
	100		3	17.5
	100		3	8.8
control	100		3	0

Mice treated with peptide ^26^[F] recovered completely after 24 h, and the survival rate was 100% after one week of observation. However, two of three individuals injected with ^26^[1-Nal] showed signs of disease and died within 8 h of peptide administration, and the remaining individual fully recovered and survived after 1 week of observation. These results suggest that the administration of both peptides via sc is safe; however, the ip inoculation of the ^26^[1-Nal] peptide showed greater toxicity than ^26^[F], survival being 33% and 100% respectively. Peptide ^26^[F] was inoculated in female mice (140 mg kg^−1^) by ip administration, after 30 min of treatment decreased motor function, abdominal contractions, excess irrigation, marked ataxia and loss of coordination were observed. After 4 h of treatment, all mice died, indicating that the lethal dose 50 (LD50) for this peptide is between 70 and 140 mg kg^−1^. While for the ^26^[1-Nal] peptide, a dose-response test was performed (table 3, experiment 2). The survival rate of mice was 100% after one week of treatment. These results are in accord with LfcinB being safe when it was administered (4 mg via intra-tumoural route) and had anti-tumoural effect when xenoinserted in NOD-SCI-gamma mice induced by human breast cancer lines MDA-MB-468 and MDA-MB-231 [27].

Toxicity studies are an essential part of preclinical evaluation and can provide valuable information on the safety of the compound, estimate the maximum tolerated dose and safe dosing guidelines. This is the first study for the determination of the maximum tolerated dose of these peptides in the murine model. Our results allowed us to establish the safe dose range of the peptide, which is relevant information for designing and developing future efficacy studies in the murine model.

### 3.4. Flow cytometry cell death type studies

**Figure 4.**
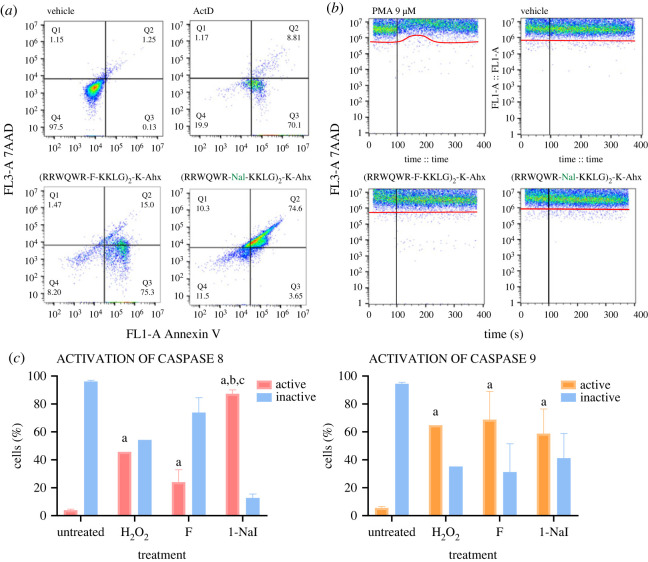
Flow cytometry assays of peptides ^26^[F] and ^26^[1-Nal]. (*a*) Annexin/7AAD assays; negative control: untreated cells. Positive control: ActD 0.5 µM. (*b*) extracellular cytosolic calcium flux assay. Negative control: vehicle. Positive control: PMA 9 µM. (*c*) Caspase 8 and 9 activation assays. Negative control: untreated cells. Positive control: H_2_O_2_. Statistically significant differences were found between cells with no treatment and the two peptides. The data represents the mean ± s.e. An independent experiment with *n* = 3 each. (ANOVA, Sidak's multiple comparisons test was used, *p* ≤ 0.05). For all assays, the peptide concentration used was the IC_50_ value determined by MTT assay.

Taking into account the previous results, the type of cell death generated by peptides ^26^[F] and ^26^[1-Nal] in the breast cancer cell line MCF-7 was studied via flow cytometry assays (figure 4). The anexin V/7AAD assays showed that peptide-treated cell populations shifted mainly towards apoptotic events. Peptide ^26^[F] primarily induced early apoptosis (Q3: 75.3%), while peptide ^26^[1-Nal] induced late apoptosis (Q2: 74.6%) (figure 4*b*). This result is quite interesting; it suggests that the change of Phe to 1-Nal in the dimeric sequence could modulate cell death towards late apoptosis.

Extracellular cytosolic calcium flux assays showed that neither peptide affected the integrity of the cytoplasmic membrane in MCF-7 cells, since there was no significant alteration in the baseline of the FL-1A channel (figure 4*a*). On the other hand, the cell death induced by both peptides is related to the activation of caspases 8 and 9. Peptide ^26^[F] primarily induces the expression of caspase 9 (70%), while peptide ^26^[1-Nal] induces activation of both caspase 8 (90%) and caspase 9 (60%). These results suggest that this peptide generates extrinsically mediated apoptosis, while ^26^[F] primarily activates intrinsically mediated apoptosis, which is consistent with results observed in annexin V/7AAD assays. The results suggest that ^26^[1-Nal] induces cell death involving several pathways simultaneously, one of these being the main one responsible for the cell death generated in the MCF-7 cell line. As observed, ^26^[1-Nal] generated an overactivation of caspase 8 which may be related to the extrinsic apoptosis pathway possibly associated with the interaction of the peptide with a membrane receptor topical yet to be studied. This could be related to the structure of the 1-Nal side chain which possesses a π-conjugated system that is related to intramolecular interactions with adjacent arginine residues through a π-cation interaction that according to recent research can change the conformation of the peptide generating recognition with membrane receptors, 1-Nal generated an overactivation of caspase 8 which may be related to the extrinsic apoptosis pathway possibly associated with the interaction of the peptide with a membrane receptor topical yet to be studied. This could be related to the structure of the 1-Nal side chain which possesses a π-conjugated system that is related to intramolecular interactions with adjacent arginine residues through a π-cation interaction that according to recent research can change the conformation of the peptide generating recognition with membrane receptors [26].

Finally, in order to determine whether dimeric peptides have a broad spectrum of action, their cytotoxic effect on the human cervical cancer cell line HeLa was evaluated. Like breast cancer, cervical cancer is of particular interest to women, as it is the fourth most frequently diagnosed cancer among women, with a mortality rate close to 56%, well above the estimated rate for breast cancer (30%) [26]. As can be seen in table 2, the three dimeric peptides had a similar concentration-dependent cytotoxic effect against HeLa cells. Peptide ^26^[F] exhibited an IC_50_ = 7 µM, while peptides ^26^[1-Nal] and ^26^[hF] exhibited an IC_50_ = 16 µM, which suggests that the three peptides have a broad spectrum of action. Interestingly, the peptide that had the greatest cytotoxic effect *in vitro* was the original peptide ^26^[F].

## 4. Conclusion

Dimeric peptides derived from peptide ^26^[F]: (RRWQWRFKKLG)_2_-K-Ahx were synthesized and characterized. The analogues with the highest synthetic viability in terms of purity and yield were ^26^[1-Nal] and ^26^[hF]. The MTT assays showed that substitution of ^26^Phe residue by Bpa, hF or 1-Nal amino acids enhance the cytotoxic effect in the MCF-7 human breast cancer cell line. Substitution of ^26^Met (IC_50_ ≥ 60 µM) for ^26^F(IC_50_ = 23 µM), ^26^hF (IC_50_ = 18 µM), ^26^1-Nal (IC_50_ = 14 µM), in the dimeric sequence (^20^RRWQWRMKKLG^30^)_2_-K-Ahx significantly increased the cytotoxic effect against MCF-7 cells, suggesting that incorporation of Phe or unnatural Phe-like amino acids at position 26 enhanced the cytotoxic effect. The possibility of making changes with different residues (Phe, hF and 1-Nal) at one position without affecting the anti-cancer activity allows to have several therapeutic options based on a template sequence. This can be useful for designing treatments to reduce resistance and increase bioavailability and versatility in drug delivery strategies, etc. In addition, peptides ^26^[F], ^26^[1-Nal] and ^26^[hF] exhibited a potent cytotoxic effect against the Hela cell line with IC_50_s of 7, 16 and 16 µM, respectively. Substitution of ^26^Phe by hF or 1-Nal at position 26 of the dimeric sequence induces resistance to proteolytic degradation caused by pepsin, while incorporation of the amino acid 1-Nal provides protection against trypsin for up to 3 h. *In vitro* cytotoxicity studies in the non-cancerous cell lines HEK-293, fibroblasts and erythrocytes showed that the peptides ^26^[F], ^26^[1-Nal] and ^26^[hF] are selective with respect to erythrocytes, and the peptide with the highest selectivity was ^26^[F], since it showed a marked therapeutic window after 100 µg ml^−1^ for all cell lines evaluated. On the other hand, preliminary toxicity studies in mice, showed that the peptides are safe at concentrations up to 140 higher than the IC_50_, which is an important result for future efficacy studies. Flow cytometry assays showed that peptides ^26^[F] and ^26^[1-Nal] did not affect cytoplasmic membrane integrity, and cell death was primarily mediated by apoptotic processes. Our results suggest that dimeric structures enhance anti-cancer activity and are more resistant to proteolytic degradation, so ^26^[F], ^26^[1-Nal] and ^26^[hF] peptides may be considered relevant for the development of cancer treatments.

## Data Availability

The supplementary material contains the analytical characterization by RP-HPLC, and MS (HRMS ESI-Q/TOF) of the six analogous peptides synthesized in this research is presented. The results are arranged in panels as follows: Panel A: structure and theoretical exact masses of the dimeric peptide. Panel B: characterization by RP-HPLC and chromatographic purity at 210 nm. Panel C: mass spectrum. The data are provided in electronic supplementary material [28].
